# Carotenoids as potential inhibitors of TNFα in COVID-19 treatment

**DOI:** 10.1371/journal.pone.0276538

**Published:** 2022-12-27

**Authors:** Farzaneh Taghipour, Nasrin Motamed, Mohammad Ali Amoozegar, Maryam Shahhoseini, Soodeh Mahdian

**Affiliations:** 1 Department of Cellular and Molecular Biology, School of Biology, College of Science, University of Tehran, Tehran, Iran; 2 Department of Microbiology, School of Biology, College of Science, University of Tehran, Tehran, Iran; 3 Department of Genetics, Reproductive Biomedicine Research Center, Royan Institute for Reproductive Biomedicine, ACECR, Tehran, Iran; 4 Reproductive Epidemiology Research Center, Royan Institute for Reproductive Biomedicine, ACECR, Tehran, Iran; 5 Department of Cellular and Molecular Biology, Faculty of Biological Sciences, North Tehran Branch, Islamic Azad University, Tehran, Iran; Alagappa University, INDIA

## Abstract

Tumor necrosis factor-alpha (TNF-α) is a multifunctional pro-inflammatory cytokine, responsible for autoimmune and inflammatory disorders. In COVID-19 patients, increased TNF-α concentration may provoke inflammatory cascade and induce the initiation of cytokine storm that may result in fatal pneumonia and acute respiratory distress syndrome (ADRS). Hence, TNFα is assumed to be a promising drug target against cytokine storm in COVID-19 patients. In the present study, we focused on finding novel small molecules that can directly block TNF-α-hTNFR1 (human TNF receptor 1) interaction. In this regards, TNF-α-inhibiting capacity of natural carotenoids was investigated in terms of blocking TNF-α-hTNFR1 interaction in COVID-19 patients with the help of a combination of *in silico* approaches, based on virtual screening, molecular docking, and molecular dynamics (MD) simulation. A total of 125 carotenoids were selected out of 1204 natural molecules, based on their pharmacokinetics properties and they all met Lipinski’s rule of five. Among them, Sorgomol, Strigol and Orobanchol had the most favorable ΔG with the best ADME (absorption, distribution, metabolism, excretion) properties, and were selected for MD simulation studies, which explored the complex stability and the impact of ligands on protein conformation. Our results showed that Sorgomol formed the most hydrogen bonds, resulting in the highest binding energy with lowest RMSD and RMSF, which made it the most appropriate candidate as TNF-α inhibitor. In conclusion, the present study could serve to expand possibilities to develop new therapeutic small molecules against TNF-α.

## Introduction

Severe acute respiratory syndrome-coronavirus-2(SARS-CoV-2) is responsible for the pandemic viral pneumonia known as COVID-19. The newly discovered COVID-19 pandemic is a major public health issue that has spread worldwide without any effective known treatment [1]. Most COVID-19 patients experience mild to moderate symptoms, however, for some patients, it is probable to run into hyper-inflammatory phase, driven by excess cytokines/chemokines production, called cytokine storm [2]. In most severe COVID-19 cases, an overwhelming immune response leads to an increased level of cytokines; such as tumor necrosis factor-α (TNF-α), interleukin-1(IL-1), interleukin-6 (IL-6), (interleukin-7) IL-7, interleukin-8 (IL-8), interleukin-17 (IL-17), which are supposed to be responsible for acute respiratory distress syndrome (ADRS), instead of viral activity [3]. Excess inflammation is known to be a critical factor in developing disease symptoms. Among cytokines that are involved in hyper-inflammation, TNF-α is o the major component of cytokine storm. As TNF-α concentration rises in the first stages of COVID-19, it is suggested as a remarkable trigger for inflammatory cascade, leading to cytokine storm that in turn results in fatal pneumonia and acute respiratory distress syndrome (ARDS) [4]. TNF-α is known to be produced in most types of inflammation and has a critical role in the development of the inflammatory response. This protein is found in the blood and tissues of infected people, functioning as an inflammation amplifier, thus its inhibition may be effective in relieving disease symptoms even in the presence of other cytokines [5]. In COVID-19 patients, lung dysfunction results from a capillary leak, which is a consequence of hyper-inflammation. Therefore, it’s assumed that, since TNF-α is important in cytokine storm initiation, its suppression can prove to be efficacious in down-regulating other pro-inflammatory mediators, including IL-1, IL-6, and granulocyte-macrophages. Studies reporting anti-IL-6 or anti-IL-1 cascade are rare [6]. Another argument for why TNF is a promising cytokine to target comes from previous studies in SARS-CoV-1 that have reported anti-TNF therapies to have protective effects on fatal SARS-CoV1 infection [7]. Additionally, it should be noted that people with immune-mediated inflammatory diseases; such as, inflammatory bowel disease (IBD) or rheumatoid arthritis and are under anti-TNF therapy, usually show decreased odds of hospitalization or intensive care [8, 9]. Regarding all these studies, it seems anti-TNF therapy can be accountable as a promising treatment for COVID-19 patients.

Tumor necrosis factor-alpha (TNF-α) is a potent pro-inflammatory cytokine, produced largely by macrophages while it can also be secreted by many other cells; such as, neutrophils, NK cells, and mast cells [10]. TNF-α is produced in response to inflammatory agents and by binding to its receptor—TNFR1—recruits other cytokines and chemokines, leading to the immune pathology, which clearly explains its remarkable role in the acute and chronic systemic inflammatory response. TNF-α is involved in a variety of autoimmune diseases including psoriasis, inflammatory bowel disease, rheumatoid arthritis, systemic sclerosis, systemic lupus erythematosus, multiple sclerosis, diabetes, and ankylosing spondylitis [11, 12]. Concerning TNF-α activity in several physiological and pathological conditions, its inhibition has been taken into consideration for the treatment of various autoimmune and inflammatory disorders [13].

A variety of anti-TNFα agents, including infliximab, etanercept, adalimumab, certolizumab and golimumab have been developed so far. However, there are limitations to their application due to their high production costs, the requirement of intravenous injection, and the possibility of potential immunogenicity [14]. There are no known small molecules that interrupt the interaction between TNF and TNFR1 [15].

In the present study, we focused on carotenoids of eukaryotic and bacterial sources, as natural compounds, to screen for novel and safe TNFα/TNFR1 inhibitors. The selection of carotenoids has been due to their diverse biological functions including antioxidant and anti-inflammatory properties and the modulation of some cellular pathways. Different in vitro and in vivo studies have reported that carotenoids have the ability to scavenge DNA damaging free radicals, suppress angiogenesis, inhibit cell proliferation and induce apoptosis. Epidemiological studies support remarkable relation between dietary intake and circulating levels of carotenoids and reduction in cancer risk/carcinoma of various organs [16]. In addition, several studies showed that apocarotenoids appear to function as anticancer factors and cellular modulators of the retinoic acid receptors, retinoid X receptors, peroxisome proliferator-activated receptors and estrogen receptors [17]. Also, There are reports that shows β-Carotene has anti-alzheimer effects on mice related to the involvement of the Nrf2/ARE pathway and lutein has anti-apoptotic effects through preventing loss of Bcl2 and Bcl-Xl, preventing accumulation of cytochrome C and accumulation of caspase 3 [18]. This study was aimed to seek for the most appropriate carotenoid candidate(s) as TNF/TNFR1 inhibitor by utilizing molecular library of carotenoids and, computational and simulation tools; such as Virtual Screening and MD simulation.

## Methods

### Preparation of proteins and ligands

The main purpose of this study was to understand the impact of carotenoids on disrupting the TNFα/TNFR1 interaction. The crystal structure of the human TNFα-TNFR1 complex has not yet been reported so far. Therefore, we used mice TNFα in complex with hTNFR1 (PDB code: 7KP7), obtained from Protein Data Bank (www.rcsb.org). The single mTNFα and hTNFR1 elements are well-arranged with previously published structures 1TNF [https://www.rcsb.org/structure/1TNF] and 1NCF [https://www.rcsb.org/structure/1NCF] [19]. The active site of mTNFα-hTNFR1 complex was figured out by using CASTp server (http://sts.bioe.uic.edu/castp/index.html?2was). CASTp identifies the protein and its receptor binding site and discovers the key amino acids that are involved in binding interactions (Glu110, Lys112, Pro113 of chain A from mTNFα, Tyr103, His105, Trp107, Gln113, Phe115, Asn116, Ser118, Asn134, Glu147 and Asn148 of chain E from hTNFR1). Finally, we used the Carotenoids Database (www.carotenoiddb.jp) to obtain intended information on carotenoids, which presents information on 1204 natural carotenoids in 722 source organisms. There is registered information available on 349 prokaryotic and 680 eukaryotic carotenoids that we included in our survey. Energy minimization analysis of selected carotenoids was carried out by PyRx and the pre-generated PDBQT files.

### Prediction of ADME parameters

In order to check small molecules for their potential activity as a drug-like agent, they must meet pharmacokinetic properties; including adsorption, distribution, metabolism and excretion (ADME), as their importance in drug discovery process has increased [20]. The depiction of ADME properties in the initial phase of drug discovery process has apparent value for successful progression in the selection of appropriate drug-like molecules with ameliorated efficacy and safety and to avoid late-stage failures [21]. For a compound to be considered as a practical drug candidate, it must be able to achieve this target in adequate concentration, and survive its functional environment for long enough to exert potential effect. The pharmacokinetics, drug-likeness, and physicochemical profile of carotenoid compounds were evaluated by the SwissADME tool. Some of the SwissADME predictions included gastrointestinal absorption by BOILED-Egg method, BBB (blood-brain barrier) permeation, interactions with Cytochrome P450 (CYP450) enzyme and being P-glycoprotein (P-gp) substrate. It is of great significance to consider if the candidate molecule interplays with CYP450 as this enzyme plays a key role in drug elimination via biotransformation, and also interacts with them to create drug-drug interactions. Another factor of importance is the potential of being P-gp substrate as this transporter plays a protective role by being involved in drug efflux, which is one of the main reasons for unsuccessful targeting of multi-drug resistant cancers that overexpress these transporters [22]. Another important aspect of drug development to be noted is drug’ solubility, one of the key factors that enormously influence the drug bioavailability. Less water-soluble drugs often have poor bioavailability, which means that a higher drug dose is required to achieve adequate concentration in systemic circulation to obtain an efficient therapeutic response. However, a higher dose of a drug with slow absorption can bring about gastrointestinal toxicity [23, 24]. Drug-likeness is determined by Lipinski’s “Rule of 5”, which specifies the degree of permeation concerning molecular weight, number of hydrogen bonds, and lipophilicity that is expressed as Log P. Small molecules with a molecular weight less than 500, 5H-bond donors, 10H-bond acceptors, and calculated log P with less than 5, are likely to have a good permeation [25]. Therefore, regarding all posed factors, we surveyed all prokaryotic and eukaryotic carotenoids to screen them based on their drug-likeness property. The filtration result showed that there are 125 carotenoids: 17 prokaryotic and 108 eukaryotic carotenoids, that have suitable features as drug candidates for molecular docking.

We also conducted the toxicity study by The Toxicity Estimation Software Tool (T.E.S.T). The Toxicity Estimation Software Tool was developed to evaluate the toxicity of chemicals using Quantitative Structure Activity Relationships (QSARs) methodologies. We used the developmental toxicity calculation to see whether our compounds cause developmental toxicity effects to humans or animals.

### Virtual Screening (VS) based on molecular docking

After filtering carotenoids, based on their ADME properties, the ones that had favorable characteristics in that exposition, entered the next procedure, employing virtual screening method to measure the capacity of lead compounds for inhibiting the target. Some preparation steps were implemented on the protein structure before docking: the water molecules were deleted from the structure, hydrogen atoms were added and hydrogen bonds got optimized. Further, the unwanted interactions were removed, and by the Gasteiger-Marsili method, partial atomic charges were discovered. In the next step, charges were appended to the complex and Kollman United Atom charges and atomic solvation parameters were attached. The complex was considered rigid while ligands were flexible. The grid map was determined based on the active site of the complex. Therefore, we set the grid box size on 45 x 43 x 46Å, and coordinates were 52, 35, -13 for x, y, and z respectively.

Molecular docking studies were implemented utilizing AutoDock Vina in PyRx 0.8 software, which is a virtual screening tool and can be exploited in the drug-discovery field for screening compound libraries against targets to gain the most favorable binding free energy for subsequent analysis [26].

### Molecular dynamics simulation

After the virtual screening procedure, 13 carotenoids were recognized to have the most appropriate binding free energy. Of which, Strigol, Sorgomol, and Orobanchol were selected in accordance with their ADME and drug-likeness properties for analyzing their structural and dynamic behavior in ligand binding to inhibit mTNFα -hTNFR1 interaction via MD Simulation.

### Installation of MD systems

MD simulations were utilized via GROMACS 5.1.4 package with gromos 54a7 force fields [1]. For system solvation, SP3 water model was utilized [2] in each simulation system. The energy minimization was done and the Normalization Visualization Tool (NVT) was ensemble for 500 ps and then the assembled NPT was utilized at 310 K. MD simulation was completed for the complexes in 100 ns. In the Molecular mechanics Poisson–Boltzmann surface area (MM-PBSA) method, the binding free energy was calculated with the g_mmpbsa tool [3]. All modeling packages were handled with the necessary quantities of chloride ions and sodium to neutralize the platform. The Periodic Boundary Condition (PBC) was used along each box’s axial direction in each modeling framework, and the SP3 water simulation was used for systemic solubilization [27].

## Results

### Virtual screening

Virtual screening was applied as a primary stage for discovering novel and potent drug-like molecules. The *in silico* ADME-PK (absorption, distribution, metabolism, excretion- pharmacokinetics) study is a fast and secure way to exploit before additional *in vitro* and *in vivo* experiments, which reduces failure rate in further steps. In the first step, Lipinski’s Rule of 5 was considered as a tool for analyzing the drug-likeness properties. Lipinski’s Rule of five could be a standard, devised for evaluating the drug-likeness, and controlling if a compound with biological and pharmacological function can have the authenticity to be applied for human use. All carotenoids, whether eukaryotic or prokaryotic, were screened with the SwissADME server to explore their drug-likeness properties. Our final results showed that 125 carotenoid ligands were safe and met the principles of Lipinski rule of five and had suitable ADME properties. Therefore, these carotenoids were selected for further studies. Pharmacokinetics properties of 125 carotenoids is presented in Table 1.

**Table 1:** Pharmacokinetic properties of 125 carotenoids with appropriate drug-likeness property.

Ligand	Source	Lipinski’s Rule	Log P	Gl absorption	BBB permeant	P-gp substrate	CYP1A2 inhibitor	CYP2C19 inhibitor	CYP2C9 inhibitor	CYP2D6 inhibitor	CYP3A4 inhibitor	ΔG
(3R)-3-Hydroxy-beta-ionone	Prokaryote	Yes; 0 violation	2.45	High	Yes	No	No	No	No	No	No	-5.5
2,4,4-trimethyl-3-(3-oxobutyl)-2-cyclohexen-1-one	Prokaryote	Yes; 0 violation	2.3	High	Yes	No	No	No	No	No	No	5.2
4-oxo-beta-Ionone	Prokaryote	Yes; 0 violation	2.31	High	Yes	No	No	No	No	No	No	-5.8
8’,10-apocarotenal	Prokaryote	Yes; 0 violation	3.1	High	Yes	No	No	Yes	Yes	No	No	-5.8
Apo-8’,15’-carotenedial	Prokaryote	Yes; 0 violation	1.85	High	Yes	No	No	No	No	No	No	-5
Apo-13-zeaxanthinone	Prokaryote	Yes, 0 violation	3.36	High	Yes	No	No	Yes	Yes	No	No	-5.9
Apo-15-zeaxanthinal	Prokaryote	Yes,0 violation	3.42	High	Yes	No	Yes	Yes	Yes	No	Yes	-6.3
beta-Apo-13-carotenone	Prokaryote	Yes, 0 violation	3.54	High	Yes	No	Yes	No	Yes	No	No	-6.2
beta-Cyclocitral	Prokaryote	Yes, 0 violation	2.23	High	Yes	No	No	No	No	No	No	-4.7
beta-Ionone	Prokaryote	Yes, 0 violation	2.75	High	Yes	No	No	No	No	No	No	-5.5
beta-Ionone-5,6-epoxide	Prokaryote	Yes, 0 violation	2.83	High	Yes	No	No	No	No	No	No	-5.6
Crocetindial	Prokaryote	Yes, 0 violation	3.79	High	Yes	No	No	Yes	Yes	No	No	-6.2
Dihydro-beta-ionol	Prokaryote	Yes, 0 violation	2.99	High	Yes	No	No	No	No	No	No	-5.2
Dihydro-beta-ionone	Prokaryote	Yes, 0 violation	2.78	High	Yes	No	No	No	No	No	No	-4.9
Tectoionols A	Prokaryote	Yes, 0 violation	2.62	High	Yes	No	No	No	No	No	No	-6.4
Tetrahydroionone	Prokaryote	Yes, 0 violation	2.79	High	Yes	No	No	No	No	No	No	-5.1
(3R)-3-Hydroxy-β-ionone	Eukaryote	Yes, 0 violation	2.45	High	Yes	No	No	No	No	No	No	-5.5
(3R,6R,7E,9R)-3,9-dihydroxy-4,7-megastigmadiene	Eukaryote	Yes, 0 violation	2.71	High	Yes	No	No	No	No	No	No	-5.7
9-Apo-6,7-didehydro-5,6-dihydro-3,5-dihydroxy-beta-carotene	Eukaryote	Yes, 0 violation	1.97	High	Yes	No	No	No	No	No	No	-5.5
(6S,7E,9E,11E)-3-Oxo-13-apo-α-caroten-13-one	Eukaryote	Yes, 0 violation	2.77	High	Yes	No	No	Yes	No	No	No	-6.1
(6S,9R)-Roseoside	Eukaryote	Yes, 0 violation	1.71	Low	No	Yes	No	No	No	No	No	-6.8
(9S)-9-Apo-7,8-dihydro-9-hydroxy-beta-caroten-4-one	Eukaryote	Yes, 0 violation	2.55	High	Yes	No	No	No	No	No	No	-5.1
(11Z)-3-Hydroxyretinal	Eukaryote	Yes, 0 violation	3.39	High	Yes	No	Yes	Yes	Yes	No	Yes	-6.6
(all-E,3R)-3-Hydroxy-α-retinal	Eukaryote	Yes, 0 violation	3.63	High	Yes	No	No	Yes	Yes	No	Yes	-6.8
2,2,6-trimethylcyclohexane-1,4-dione	Eukaryote	Yes, 0 violation	1.63	High	Yes	No	No	No	No	No	No	-4.8
2,2,6-trimethylcyclohexanone	Eukaryote	Yes, 0 violation	2.18	High	Yes	No	No	No	No	No	No	-4.6
2,5-Epoxy-megastigma-6(Z),8(E)-diene	Eukaryote	Yes, 0 violation	3.1	High	Yes	No	No	No	Yes	No	No	-5.4
2-hydroxy-2,6,6-trimethylcyclohexanone	Eukaryote	Yes, 0 violation	1.85	High	Yes	No	No	No	No	No	No	-5
3,9-Dihydroxy-4-megastigmene	Eukaryote	Yes, 0 violation	2.63	High	Yes	No	No	No	No	No	No	-5.3
3-Hydroxy-13-apo-α-caroten-13-one	Eukaryote	Yes, 0 violation	3.34	High	Yes	No	No	Yes	Yes	No	No	-6.1
3-Hydroxy-beta-homocyclocitral	Eukaryote	Yes, 0 violation	1.97	High	Yes	No	No	No	No	No	No	-4.9
3-Hydroxy-α-Ionone	Eukaryote	Yes, 0 violation	2.44	High	Yes	No	No	No	No	No	No	-5.4
3-Hydroxy-β-damascone	Eukaryote	Yes, 0 violation	2.43	High	Yes	No	No	No	No	No	No	-5.4
3-OH-beta-Cyclocitral;	Eukaryote	Yes, 0 violation	1.86	High	Yes	No	No	No	No	No	No	5.2
3-oxo-α-Ionol	Eukaryote	Yes, 0 violation	1.98	High	Yes	No	No	No	No	No	No	-5.9
3β-hydroxy-5α,6α-epoxy-7-megastigmen-9-one	Eukaryote	Yes, 0 violation	2.43	High	Yes	No	No	No	No	No	No	-5.5
4-deoxyorobanchol	Eukaryote	Yes, 0 violation	3.2	High	Yes	No	No	No	Yes	No	No	-7.6
4-hydroxy-2,6,6- trimethylcyclohexanone	Eukaryote	Yes, 0 violation	1.81	High	Yes	No	No	No	No	No	No	-5.2
4-hydroxy-2,6,6-trimethyl-2-cyclohexen-l-one	Eukaryote	Yes, 0 violation	1.8	High	Yes	No	No	No	No	No	No	-5.2
4-Oxoretinaldehyde	Eukaryote	Yes, 0 violation	3.5	High	Yes	No	Yes	Yes	Yes	No	No	-6.4
4-oxo-β-ionone	Eukaryote	Yes, 0 violation	2.31	High	Yes	No	No	No	No	No	No	-5.8
5,6-Epoxy-3-hydroxy-β-ionone	Eukaryote	Yes, 0 violation	2.56	High	Yes	No	No	No	No	No	No	-6
5-Deoxystrigol	Eukaryote	Yes, 0 violation	3.21	High	Yes	No	No	No	Yes	No	No	-7.4
9-Apo-7,8-dihydro-3,9-dihydroxy-beta-carotene	Eukaryote	Yes, 0 violation	2.8	High	Yes	No	No	No	No	No	No	-5
9-Apo-7,8-dihydro-3-hydroxy-beta-caroten-9-one	Eukaryote	Yes, 0 violation	2.47	High	Yes	No	No	No	No	No	No	-5.2
10-apo-19-nor-6,7-didehydro-epsilon-carotn-3,9-dione	Eukaryote	Yes, 0 violation	2.24	High	Yes	No	Yes	No	No	No	No	-5.1
10-apo-19-nor-7,8-didehydro-epsilon-carotn-3,9-dione	Eukaryote	Yes, 0 violation	2.37	High	Yes	No	No	No	No	No	No	-5.5
12,12’-Diapocarotene-dial	Eukaryote	Yes, 0 violation	1.8	High	Yes	No	No	No	No	No	No	-4.9
19-Hydroxy-carlactone	Eukaryote	Yes, 0 violation	3.28	High	Yes	No	No	Yes	Yes	No	No	-6.1
Abscisic alchol	Eukaryote	Yes, 0 violation	2.44	High	Yes	No	No	No	No	No	No	-6
Aeginetin	Eukaryote	Yes, 0 violation	3.82	High	No	Yes	No	Yes	Yes	No	Yes	-6.8
alpha-cyclocitral	Eukaryote	Yes, 0 violation	2.23	High	Yes	No	No	No	No	No	No	-4.8
alpha-Ionone	Eukaryote	Yes, 0 violation	2.81	High	Yes	No	No	No	Yes	No	No	-5.5
Annuionone A	Eukaryote	Yes, 0 violation	2.13	High	Yes	No	No	No	No	No	No	-5.5
Annuionone B	Eukaryote	Yes, 0 violation	2.05	High	Yes	No	No	No	No	No	No	-5.7
Annuionone C	Eukaryote	Yes, 0 violation	2.38	High	Yes	No	No	No	No	No	No	-5.4
Annuionone D	Eukaryote	Yes, 0 violation	2.28	High	Yes	No	No	No	No	No	No	-5.5
Annuionone E	Eukaryote	Yes, 0 violation	2.36	High	Yes	No	No	No	No	No	No	-6.3
Annuionone F	Eukaryote	Yes, 0 violation	1.86	High	No	No	No	No	No	No	No	-5.4
Annuionone H	Eukaryote	Yes, 0 violation	2.41	High	Yes	No	No	No	No	No	No	-6
Apo-8’-bixinal	Eukaryote	Yes, 0 violation	4.67	High	Yes	Yes	No	No	Yes	No	No	-5.4
Apo-10’-fucoxanthinal	Eukaryote	Yes, 0 violation	4.35	High	No	Yes	Yes	Yes	Yes	No	Yes	-7.2
Apo-10’-violaxanthal	Eukaryote	Yes, 0 violation	4.78	High	No	Yes	Yes	Yes	Yes	No	Yes	-6.7
Apo-11-zeaxanthinal	Eukaryote	Yes, 0 violation	2.61	High	Yes	No	No	No	No	No	No	-5.7
Apo-12’-capsorubinal	Eukaryote	Yes, 0 violation	4.21	High	Yes	Yes	Yes	Yes	Yes	No	Yes	-6.8
Apo-12’-fucoxanthinal	Eukaryote	Yes, 0 violation	4.04	High	Yes	Yes	Yes	Yes	Yes	Yes	Yes	-6.9
Apo-12’-violaxanthal	Eukaryote	Yes, 0 violation	4.45	High	Yes	Yes	No	Yes	Yes	No	Yes	-7.1
Apo-13-zeaxanthinone	Eukaryote	Yes, 0 violation	3.36	High	Yes	No	No	Yes	Yes	No	No	-6
Apo-14’-zeaxanthinal	Eukaryote	Yes, 0 violation	3.87	High	Yes	Yes	Yes	Yes	Yes	No	Yes	-6.7
Apo-15-zeaxanthinal	Eukaryote	Yes, 0 violation	3.92	High	Yes	No	Yes	Yes	Yes	No	Yes	-6.4
Azafrinaldehyde	Eukaryote	Yes, 0 violation	4.73	High	No	Yes	Yes	Yes	Yes	No	Yes	-6.3
Azafrin	Eukaryote	Yes, 0 violation	4.47	High	No	Yes	No	Yes	Yes	No	Yes	-7
beta-Cyclocitral	Eukaryote	Yes, 0 violation	2.23	High	Yes	No	No	No	No	No	No	-4.7
beta-homocyclocitral	Eukaryote	Yes, 0 violation	2.26	High	Yes	No	No	No	No	No	No	-4.6
Blumenol A	Eukaryote	Yes, 0 violation	2.06	High	Yes	No	No	No	No	No	No	-6
Blumenol B	Eukaryote	Yes, 0 violation	2.24	High	Yes	No	No	No	No	No	No	-5.8
Blumenol C	Eukaryote	Yes, 0 violation	2.54	High	Yes	No	No	No	No	No	No	-5.3
Boscialin	Eukaryote	Yes, 0 violation	2.29	High	Yes	No	No	No	No	No	No	-5.4
C9-aldehyde	Eukaryote	Yes, 0 violation	1.54	High	Yes	No	No	No	No	No	No	-5.8
C13-spiroether theaspirone	Eukaryote	Yes, 0 violation	2.56	High	Yes	No	No	No	No	No	No	-5.6
C14 aldehyde	Eukaryote	Yes, 0 violation	2.49	High	Yes	No	No	Yes	No	No	No	-5.4
C27 epoxy-apocarotenal	Eukaryote	Yes, 0 violation	4.49	High	Yes	Yes	No	Yes	Yes	No	Yes	-6.9
Carlactone	Eukaryote	Yes, 0 violation	3.73	High	Yes	No	Yes	Yes	Yes	No	Yes	-6.8
Carlactonoic acid	Eukaryote	Yes, 0 violation	3.03	High	Yes	No	No	Yes	Yes	No	No	-6.4
Crocetindial	Eukaryote	Yes, 0 violation	3.71	High	Yes	No	No	Yes	Yes	No	No	-6.1
Crocusatin H	Eukaryote	Yes, 0 violation	1.11	High	No	No	No	No	No	No	No	-5.4
Cyclohexenone	Eukaryote	Yes, 0 violation	2.9	High	No	Yes	No	No	No	No	No	-6.8
Dehydrovomifoliol	Eukaryote	Yes, 0 violation	2.1	High	Yes	No	No	No	No	No	No	-5.9
F348	Eukaryote	Yes, 0 violation	4.89	High	No	Yes	No	Yes	Yes	No	Yes	-7.4
Geranylacetone	Eukaryote	Yes, 0 violation	3.21	High	Yes	No	No	No	No	No	No	-5.3
Grasshopper ketone	Eukaryote	Yes, 0 violation	1.97	High	Yes	No	No	No	No	No	No	-5.5
Loliolide	Eukaryote	Yes, 0 violation	2.01	High	Yes	No	No	No	No	No	No	-5.8
Megastigma-4,7E,9-trien-3-one	Eukaryote	Yes, 0 violation	2.7	High	Yes	No	No	No	No	No	No	-5.4
Megastigma-5,8-dien-4-one	Eukaryote	Yes, 0 violation	2.78	High	Yes	No	No	No	No	No	No	-5.1
Methyl carlactonoate	Eukaryote	Yes, 0 violation	3.73	High	Yes	No	Yes	Yes	Yes	No	No	-6.8
Mycorradicin	Eukaryote	Yes, 0 violation	2.11	High	Yes	No	No	Yes	No	No	No	-5.8
Orobanchol	Eukaryote	Yes, 0 violation	2.9	High	No	No	No	No	No	No	No	-7.1
Oxoedulan	Eukaryote	Yes, 0 violation	2.57	High	Yes	No	No	No	No	No	No	-5.6
Oxygenated theaspirane derivative	Eukaryote	Yes, 0 violation	2.9	High	Yes	No	No	No	No	No	No	-5.8
Persicachrome	Eukaryote	Yes, 0 violation	4.65	High	Yes	Yes	No	Yes	Yes	No	Yes	-7.2
Persicaxanthin	Eukaryote	Yes, 0 violation	4.87	High	Yes	Yes	No	Yes	Yes	Yes	Yes	-6.8
Picrocrocin	Eukaryote	Yes, 0 violation	1.8	High	No	Yes	No	No	No	No	No	-6.6
Plinol A	Eukaryote	Yes, 0 violation	2.5	High	Yes	No	No	No	No	No	No	-4.6
Pseudoionone	Eukaryote	Yes, 0 violation	3.08	High	Yes	No	No	No	No	No	No	-5.1
Retro-C18-dione	Eukaryote	Yes, 0 violation	3.57	High	Yes	No	No	Yes	Yes	No	No	-4.9
Rosafluin	Eukaryote	Yes, 0 violation	2.97	High	Yes	No	No	No	No	No	No	-5.4
safranal	Eukaryote	Yes, 0 violation	2.13	High	Yes	No	No	No	No	No	No	-4.8
Sinensiaxanthin	Eukaryote	Yes, 0 violation	4.96	High	No	Yes	No	Yes	Yes	No	Yes	-7.2
Sorgolactone	Eukaryote	Yes, 0 violation	3.12	High	Yes	No	No	No	Yes	No	No	-7.2
Sorgomol	Eukaryote	Yes, 0 violation	2.96	High	No	No	No	No	No	No	No	-7
Strigol	Eukaryote	Yes, 0 violation	2.89	High	No	No	No	No	No	No	No	-7.6
Strigyl acetate	Eukaryote	Yes, 0 violation	3.08	High	No	No	No	No	Yes	No	No	-7.1
Sulcatone	Eukaryote	Yes, 0 violation	2.23	High	Yes	No	No	No	No	No	No	-4.4
Tabanone	Eukaryote	Yes, 0 violation	2.74	High	Yes	No	No	No	No	No	No	-5.5
Tectoionol derivative	Eukaryote	Yes, 0 violation	1.76	High	Yes	No	No	No	No	No	No	-6.1
Tectoionols A	Eukaryote	Yes, 0 violation	2.62	High	Yes	No	No	No	No	No	No	-6.4
Tectoionols B	Eukaryote	Yes, 0 violation	2.73	High	Yes	No	No	No	No	No	No	-5.2
Theaspirane	Eukaryote	Yes, 0 violation	2.95	High	Yes	No	No	No	Yes	No	No	-5.7
Valenciaxanthin	Eukaryote	Yes, 0 violation	5.12	High	No	Yes	No	Yes	Yes	No	Yes	-7.1
Vitamin A3	Eukaryote	Yes, 0 violation	3.88	High	Yes	No	No	Yes	Yes	Yes	No	-6.5
Vitispirane	Eukaryote	Yes, 0 violation	2.86	High	Yes	No	No	No	Yes	No	No	-5.8
β-Apo-13-carotenone	Eukaryote	Yes, 0 violation	3.54	High	Yes	No	Yes	No	Yes	No	No	-6.1
β-Damascenone	Eukaryote	Yes, 0 violation	2.74	High	Yes	No	Yes	No	No	No	No	-6.1
β-Damascone	Eukaryote	Yes, 0 violation	2.81	High	Yes	No	No	No	No	No	No	-6
γ-Damascenone	Eukaryote	Yes, 0 violation	2.58	High	Yes	No	No	No	Yes	No	No	-5.2

Toxicity of carotenoids was also evaluated by T.E.S.T software. The developmental toxicity endpoint calculation was selected to observe what is carotenoids’ toxicity effect on humans and animal. It predicted the activity/inactivity of compounds by endpoint values in such a way that if the calculated score is <0.5 then activity is negative, and if calculated score is > = 0.5 then activity is positive. The results are presented as S1 Table.

### Molecular docking

**Fig 1:**
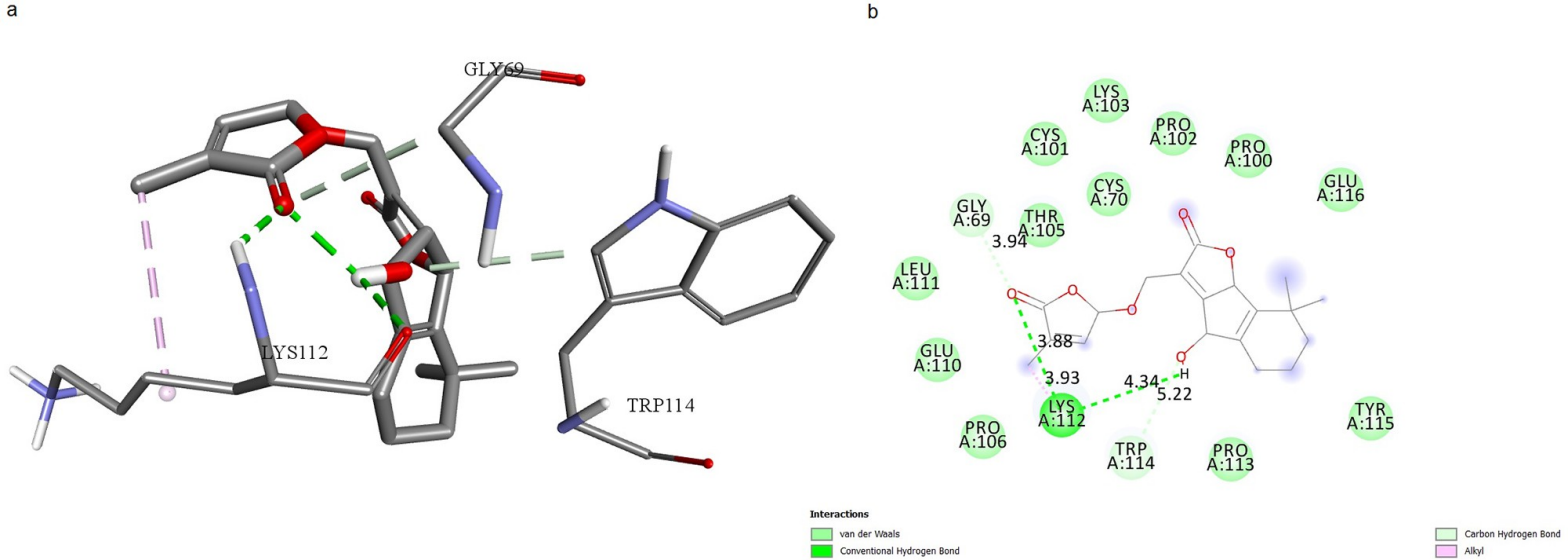
mTNFα-hTNFR1 interaction with Orobanchol. **a**. 3D shape of mTNFα-hTNFR1 interaction with Orobanchol. **b**. Illustration of hydrophobic bond formation between Orobanchol and the mTNFα-hTNFR1 complex. Orobanchol forms 2 hydrogen bonds with LYS112 of mTNFα.

**Fig 2:**
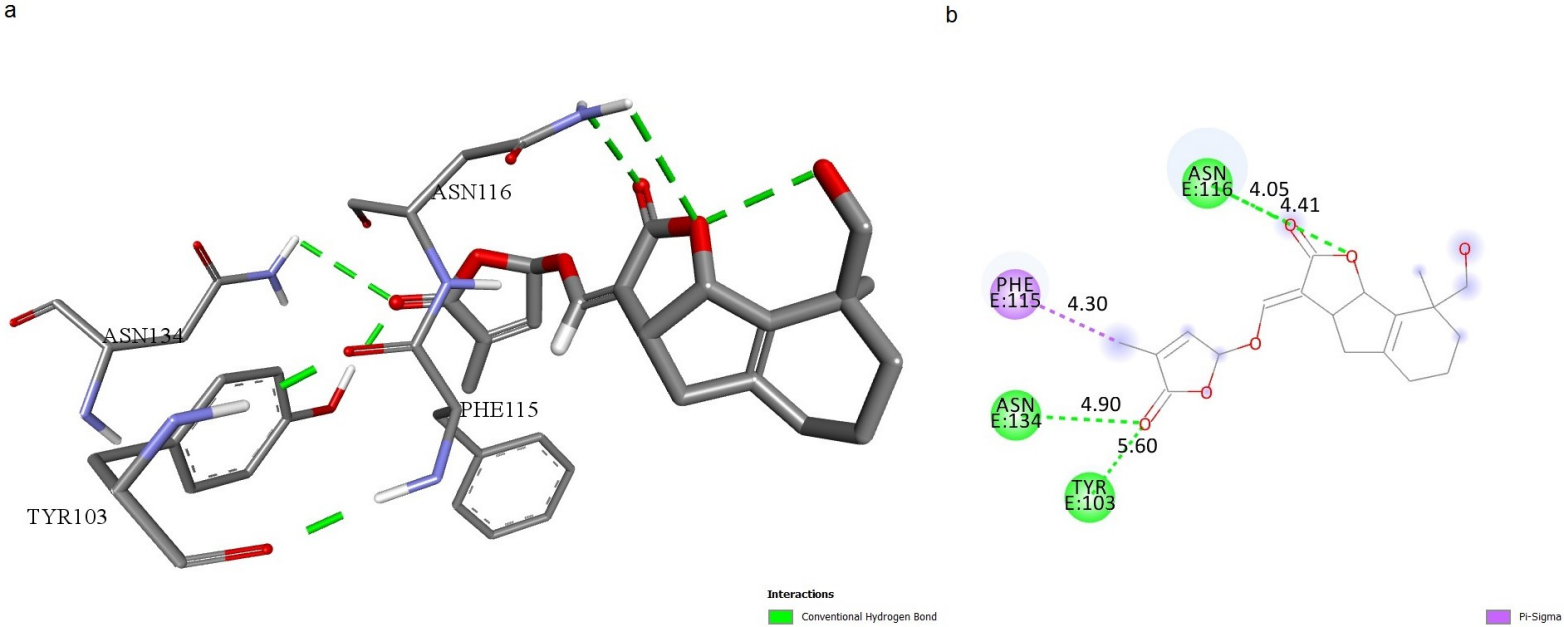
mTNFα-hTNFR1 interaction with Strigol. **a**.3D shape of mTNFα-hTNFR1 interaction with Strigol. **b**. Illustration of hydrophobic bond formation between Strigol and the mTNFα-hTNFR1 complex. Strigol forms 3 hydrogen bonds with ASN116, ASN134, and TYR103, and 1 π-sigma bond with PHE115 of hTNFR1.

**Fig 3:**
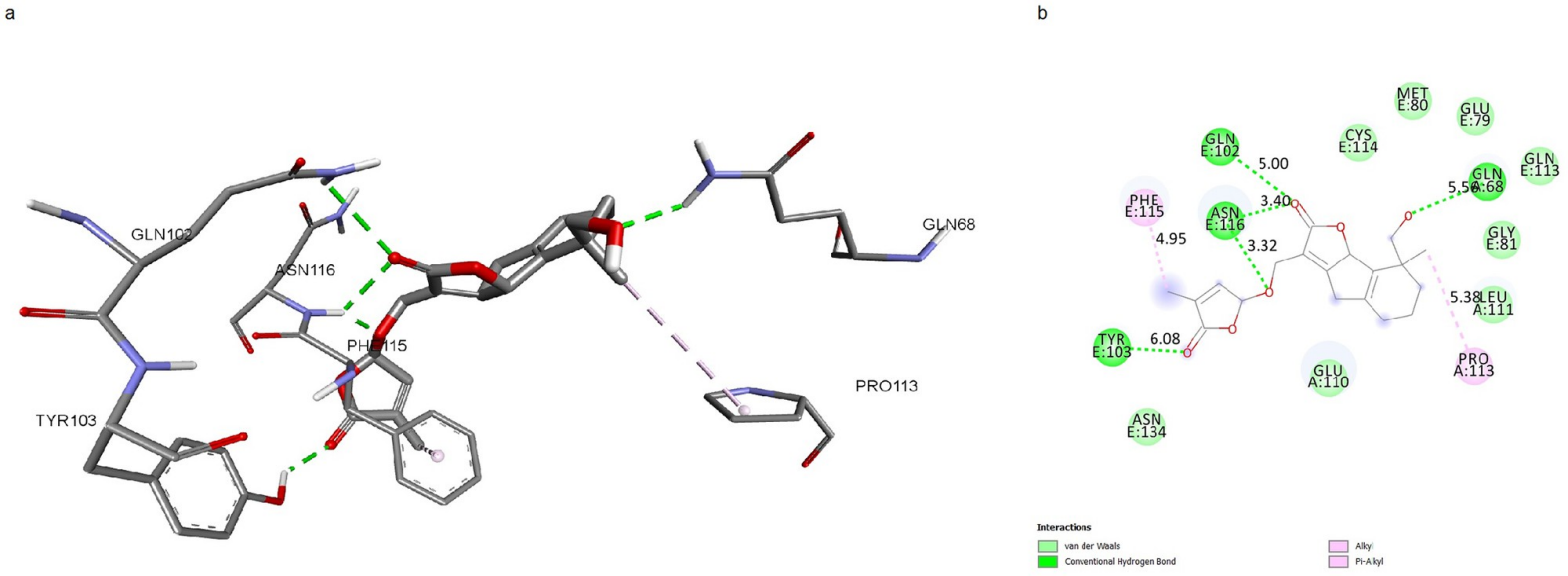
mTNFα-hTNFR1 interaction with Sorgomol. **a**.3D shape of mTNFα-hTNFR1 interaction with Sorgomol. **b**. Illustration of hydrophobic bond formation between Sorgomol with the mTNFα-hTNFR1 complex. Sorgomol forms 3 hydrogen bonds with GLN102, ASN116, and TYR103, and 1 π-sigma bond with PHE115 of hTNFR1. It also forms 1 hydrogen bond with GLN68, and 1 π-sigma bond with PRO113 of mTNFα.

Molecular docking studies were used to predict the different ligand binding modes to a proper protein binding site. Molecular docking was carried out to screen 125 carotenoids that were filtered by Lipinski’s Rule of 5. Molecular docking analysis was done using AutoDock Vina with PyRx 0.8. The selected carotenoids were docked into the binding pocket of TNF protein. Among 125 selected carotenoids, the ones with the most appropriate ΔG in interaction with mTNFα-hTNFR1 complex were specified by molecular docking. A total of 13 carotenoids were revealed to have ΔG less than -7 and were selected to be considered for additional studies. The docking results of the carotenoids with the most favorable ΔG are shown in Table 2. Eventually, 3 carotenoids were selected, based on their ideal ADME properties including solubility, high absorption, not being Pg-p substrate, and not inhibiting CYP enzymes; as well as the good docking scores and appropriate binding mode as potential drug-like molecules for molecular dynamics. Docking results showed that the top 3 ligands had interactions with residues, located in the active site of TNF, and indicated a high affinity between -7 and -7.6 kcal/mol. The number of hydrogen bonds, studied via Discovery Studio is shown in Figs 1–3.

**Table 2:** Carotenoids with the most favorable ΔG value.

Ligand	Source	Lipinski’s Rule	Log P	Gl absorption	Pg-p substrate	CYP1A2 inhibitor	CYP2C19 inhibitor	CYP2C9 inhibitor	CYP2D6 inhibitor	CYP3A4 inhibitor	ΔG
4-deoxyorobanchol	Eukaryote	Yes, 0 violation	3.2	High	No	No	No	Yes	No	No	-7.6
5-Deoxystrigol	Eukaryote	Yes, 0 violation	3.21	High	No	No	Yes	No	No	No	-7.4
Apo-10’-fucoxanthinal	Eukaryote	Yes, 0 violation	4.35	High	Yes	Yes	Yes	Yes	No	Yes	-7.2
Apo-12’-violaxanthal	Eukaryote	Yes, 0 violation	4.45	High	Yes	No	Yes	Yes	No	Yes	-7.1
F348	Eukaryote	Yes, 0 violation	4.89	High	Yes	No	Yes	Yes	No	Yes	-7.4
Orobanchol	Eukaryote	Yes, 0 violation	2.9	High	No	No	No	No	No	No	-7.1
Persicachrome	Eukaryote	Yes, 0 violation	4.87	High	Yes	No	Yes	Yes	No	Yes	-7.2
Sinensiaxanthin	Eukaryote	Yes, 0 violation	4.96	High	Yes	No	Yes	Yes	No	Yes	-7.2
Sorgolactone	Eukaryote	Yes, 0 violation	3.12	High	No	No	No	Yes	No	No	-7.2
Sorgomol	Eukaryote	Yes, 0 violation	2.96	High	No	No	No	No	No	No	-7
Strigol	Eukaryote	Yes, 0 violation	2.89	High	No	No	No	No	No	No	-7.6
Strigyl acetate	Eukaryote	Yes, 0 violation	3.08	High	No	No	No	Yes	No	No	-7.1
Valenciaxanthin	Eukaryote	Yes, 0 violation	5.12	High	Yes	No	Yes	Yes	No	Yes	-7.1

### Molecular dynamics simulation

Molecular Dynamics simulation is a valuable tool for the inspection of binding interactions between protein and its inhibitors. MD simulations were carried out for 3 lead compounds, which were studied to observe the energy and structural changes in the active site of TNF protein against its inhibitors. It reflects the general stability of the complex during a 100 nanoseconds simulation run.

#### RMSD & RMSF

**Fig 4:**
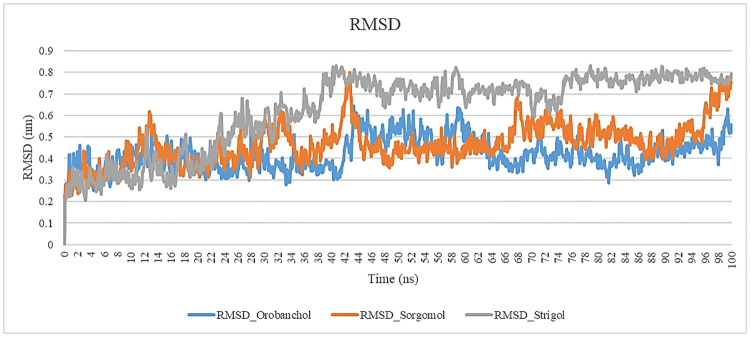
Root mean square deviation (RMSD) of c-alpha for the protein in interaction with Sorgomol, Orobanchol and Strigol during 100 ns simulation.

Parameters such as root-mean-square deviation (RMSD) and root-mean-square-fluctuation (RMSF) were studied to recognize the proteins’ stability and conformational transformation during 100 ns simulation. The RMSD plot shows the changes of backbone atoms in a protein as compared to its initial conformation. The lower RMSD implies more sustainability in the complex through ligand interaction. Knowledge, related to the conformational stability of the complexes, was estimated by evaluation of the c-alpha RMSD [26]. The RMSD of the three carotenoids and complex is depicted in Fig 4. The RMSD values of protein for Sorgomol are less than that of Strigol and Orobanchol.

**Fig 5:**
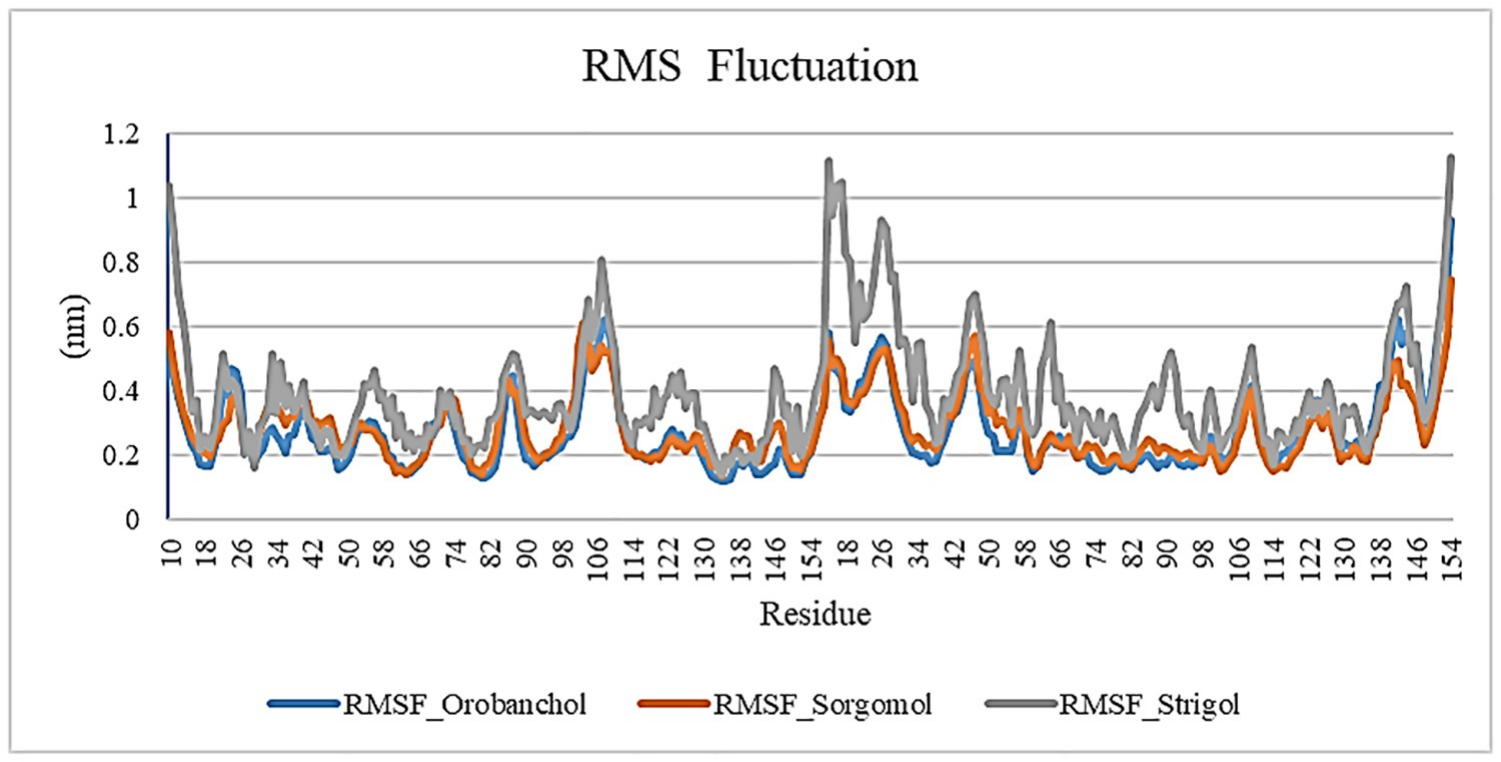
Root mean square fluctuation (RMSF) of c-alpha for the protein in interaction with Sorgomol, Orobanchol and Strigol during 100 ns simulation.

RMSF analysis helps understand the modification of protein’s amino acids during the trajectory. RMSF analysis discovers the effect of ligand-binding on the stability of protein structure over the 100ns MD simulation. RMSF of TNF backbone atoms were examined and calculated by MD trajectories in each system. A high value of RMSF shows more flexibility, while a low RMSF value shows restricted shifting and more steadiness. Residues with lower RMSF values are more stable because of the restricted movements during simulations. The RMSF study shows how the ligand binding impacts protein residues. The RMSF values are depicted in Fig 5. Analysis of RMSF indicates that the protein interaction with Sorgomol has the less flexibility than Orobanchol and Strigol throughout the course.

#### The free energy of interaction

The binding energy of three ligands and protein, ΔG, was calculated for polar and nonpolar interactions and is shown in Table 3. Sorgomol binding energy to the TNFα-TNFR1 was– 103.48, which was more favorable than Strigol and Orobanchol. With regards to the results, Sorgomol seems to have a higher affinity for protein binding.

**Table 3:** Calculation of binding free energy between TNFα-TNFR1 with orobanchol, sorgomol and strigol.

Energetic analysis of TNFα-TNFR1 / Orobanchol binding (kcal/mol)	
Van der waals energy	-88.758 +/- 35.429 kJ/mol
Electrostatic energy	-41.912 +/- 35.708 kJ/mol
Polar solvation energy	59.858 +/- 36.866 kJ/mol
SASA energy	-9.654 +/- 3.862 kJ/mol
SAV energy	0.000 +/- 0.000 kJ/mol
WCA energy	0.000 +/- 0.000 kJ/mol
Binding energy	-80.466 +/- 42.684 kJ/mol
Energetic analysis of TNFα-TNFR1 / Sorgomol binding (kcal/mol)	
Van der waals energy	-100.452 +/- 33.995 kJ/mol
Electrostatic energy	-30.791 +/- 21.426 kJ/mol
Polar solvation energy	39.064 +/- 20.477 kJ/mol
SASA energy	-10.869 +/- 3.615 kJ/mol
SAV energy	0.000 +/- 0.000 kJ/mol
WCA energy	0.000 +/- 0.000 kJ/mol
Binding energy	-103.048 +/- 36.602 kJ/mol
Energetic analysis of TNFα-TNFR1 / Strigol binding (kcal/mol)	
Van der waals energy	-124.861 +/- 37.056 kJ/mol
Electrostatic energy	-40.756 +/- 23.135 kJ/mol
Polar solvation energy	50.652 +/- 17.455 kJ/mol
SASA energy	39.316 +/- 6.274 kJ/mol
SAV energy	0.000 +/- 0.000 kJ/mol
WCA energy	0.000 +/- 0.000 kJ/mol
Binding energy	-75.649 +/- 39.764 kJ/mol

## Discussion

In the process of finding novel and natural inhibitors for TNF, combining ADME virtual screening with docking has proved to be useful in exploring the mechanism of ligand binding to the TNF pocket and calculating the affinity of such bonds using a scoring function. Moreover, molecular dynamics can be applied to improve the structures of the final complexes from docking, estimate interaction energies in a more detailed way, and come up with information about the ligand-binding mechanism and binding free energy calculations [28].

The binding of TNFα to its receptor, hTNF1, plays an important role in the initiation of inflammatory cascades in COVID-19, which can lead to cytokine storm that is responsible for ADRS [4]. Accordingly, previous reports state that the COVID-19 patients, who have been on anti-TNFα therapy, show lesser disease severity and fewer hospitalization cases [8, 9]. So we studied the impact of carotenoids in disruption of this interaction by their direct binding to TNFα. To date, no potential small-molecule therapeutics have been developed so far to block the high-affinity TNF-TNFR interaction [19]. Therefore, the present study attempted to recognize novel small-molecule inhibitors that can interrupt TNFα -TNFR1 interaction. Picking out carotenoids as natural compounds for this study is because of their various biological functionality in molecular pathways, their anti-inflammatory role, and their unique antioxidant effects. The primary selection of carotenoids was performed via the criteria including drug-like properties, and Lipinski’s “rule of five”. After that, the compounds were analyzed through molecular docking using PyRx software. Among the potential drug-like carotenoids, the ones with the most appropriate ΔG in interaction with mTNFα -hTNFR1 complex were specified by virtual screening. The top 13 carotenoids with high docking scores, low RMSD, and appropriate binding modes were specified as potential drug candidates for further studies using molecular dynamics simulations. Between the carotenoids with the best binding free energy, we opted 3 carotenoids that were assessed to have ideal pharmacokinetic properties to promise a high degree of effectiveness and safety. We assessed the binding affinity between ligands and mTNFα, aiming to block mTNFα binding to its receptor, by calculating the ΔG utilization during 100 ns. Sorgomol binding energy was recorded to be– 103.48, and had more favorable binding energy than Strigol and Orobanchol. Concerning the results, Sorgomol seemed to have a higher affinity for protein binding.

We predicted the active site of the complex using CASTp server to investigate ligands’ potential to form bonds with active-site residues, resulting in blocking the mTNFα-hTNFR1 interaction (Glu110, Lys112, Pro113 of chain A from mTNFα, Tyr103, His105, Trp107, Gln113, Phe115, Asn116, Ser118, Asn134, Glu147, Asn148 of chain E from hTNFR1). We surveyed the ligands’ binding mode of interactions with the complex. Orobanchol formed 2 hydrogen bonds with LYS112, located in the active site of mTNFα (Fig 1). The length of bonds is 3.93 and 4.34 Å. Strigol interacted with the active-site residues ASN116, ASN134, TYR103 of the protein, through hydrogen bond formation with bond length of 4.05, 4.9, 5.6 Å respectively, and with PHE115 through π-sigma bond formation with bond length of 4.3 Å (Fig 2). Sorgomol showed interactions with both the residues of the protein and its receptor. It interacted with PRO113 residue of protein through a π-Alkyl bond with bond length of 4.95 Å, and GLN68 via hydrogen bond with bond length of 5.5 Å. Besides those interactions, Sorgomol created interactions with receptor’s PHE115 residues through π-Alkyl bond with 4.95 Å bond length, and GLN102, ASN116, and TYR103 through hydrogen bonds, respectively (Fig 3). It formed 2 hydrogen bonds via ASN116 with bond length of 3.32 and 3.4 Å. Among the residues with which Sorgomol formed a bond, GLN68 and GLN102 weren’t located in the active site. However, the other bonds with the residues in active site had the ability to disrupt mTNFα-hTNFR1interaction. The results have revealed that Sorgomol interactions with residues of both the protein and its receptor, contribute to a higher binding free energy and lower RMSD value, which makes this ligand to be considered as a competent inhibitor for interrupting the interaction between mTNFα-hTNFR1.

The total amount of MM/PBSA binding free energy was estimated by calculation of nonpolar and polar interactions including van der waals ener, electrostatic, polar solvation and SASA energy. The free energy of interaction showed the binding energy of Sorgomol, Strigol and Orobanchol was -103.48, -75.649, and -80.466 kJ/mol respectively. Therefore, Sorgomol has the most appropriate binding energy in comparison with Strigol and Orobanchol, and seems to have a higher affinity for binding to the complex.

McMillan et al. [19] reported some potent small-molecule inhibitors that were able to preserve distorted TNF trimer, so that the TNFR1 affinity was decreased leading to receptor binding from three to two for each TNF trimer. As TNF concentration rises, it is more likely for the third receptor to bind the TNF, however, such binding to a distorted protein results in an irregular signaling pathway. It explains why inhibitors become less successful as TNF concentration increases. In another study, O’Connell et al. [15] reported some orally active small molecules that are able to diminish TNF signaling via TNFR1 by transforming TNF conformation and stabilizing its distorted form, resulting in decreased affinity of TNFR1. They analyzed small molecules, which block TNF activity both in vitro and in vivo. In the end, they brought up the superiority of small molecules over biologics, regarding their ability to diffuse across the blood-brain barrier, shorter half-lives, painless oral delivery, and lower cost. In another study, Saddala et al. [12] performed pharmacophore modeling and reported 15 lead potent compounds, which met all the standards to be served as novel inhibitors for TNFα, TNFR1 and TNFα-TNFR1 complex. In one study related to carotenoids and COVID-19, A Khalil et al. [29] reported carotenoids may have functions as immune-enhancers against COVID-19 by targeting the inflammatory storm resulting from viral infections. Thus, it is suggested further research and clinical trials on carotenoids therapeutic strategies. In another recent study by S Mujwar et al. [30] conducted by molecular docking and MD simulation, it is supported that 20 food-derived carotenoids have strong binding affinity with significant inhibitory effects on different target proteins of SARS-CoV-2 including replication complex, helicase, ADRP, and S protein. These studies draw more attention for further investigations on carotenoids as therapeutic inhibitors.

Identifying small molecules as inhibitors of TNFα-TNFR1 interaction has become an appealing approach for the treatment of inflammatory diseases. Recent studies have focused upon recognizing small molecules that can interrupt TNFα-TNFR1 interaction by directly binding to it. In this study, we assessed carotenoids’ inhibitory potential by *in silico* ADME studies, docking and MD simulation. Considering the diverse and significant biological functions of carotenoids, it was worth-noting to screen them by ADME studies filtration. Our study enlightens a new perspective for carotenoids as natural compounds by providing a throughout library on carotenoids drug-likeness property which can be executed for a variety of different purposes such as inhibiting other cellular pathways., In addition, the present study investigated carotenoids potential on inhibition of TNFα-TNFR1 interaction which highlight the importance of exploration for novel and capable small molecules as inhibitors of this interaction. In Conclusion, natural preventive therapeutic approaches from bioactive compounds such as carotenoids, should be assessed for establishment of new drugs against important biological interactions.

## Supporting information

S1 Table Toxicity of carotenoids. Carotenoids’ toxicity was calculated by T.E.S.T software.
(XLSX)

S2 Table Pharmacokinetics properties of carotenoids. Carotenoids’ pharmacokinetics properties were evaluated by SwissADME tool.
(XLSX)

S3 Table Docking results of carotenoids. Molecular docking was performed on carotenoids and results are presented in the table.
(XLSX)

S1 File 3D structure of 13 selected carotenoids. 13 carotenoids were selected based on docking results and with the most appropriate pharmacokinetics properties.
(DOCX)
