# Supplementary material for: Carotenoids as potential inhibitors of TNFα in COVID-19 treatment
Source: PLoS One. 2022 Dec 27;17(12):e0276538. doi: 10.1371/journal.pone.0276538 (PMC9794061; doi:10.1371/journal.pone.0276538)

3D structure of 13 selected carotenoids

4-deoxyorobanchol


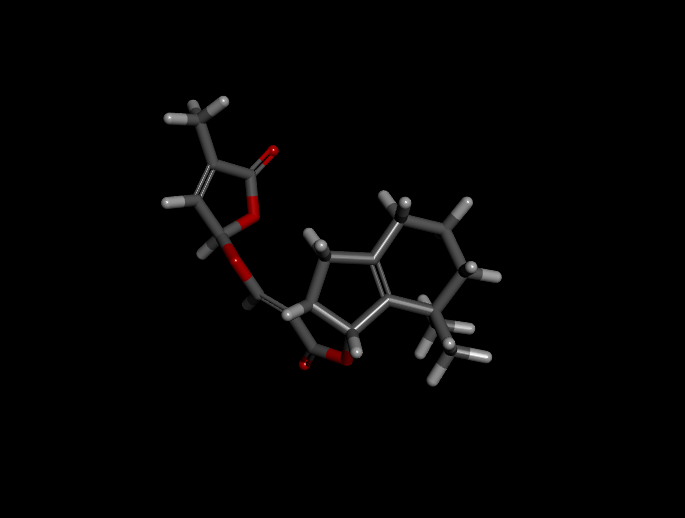


5-Deoxystrigol


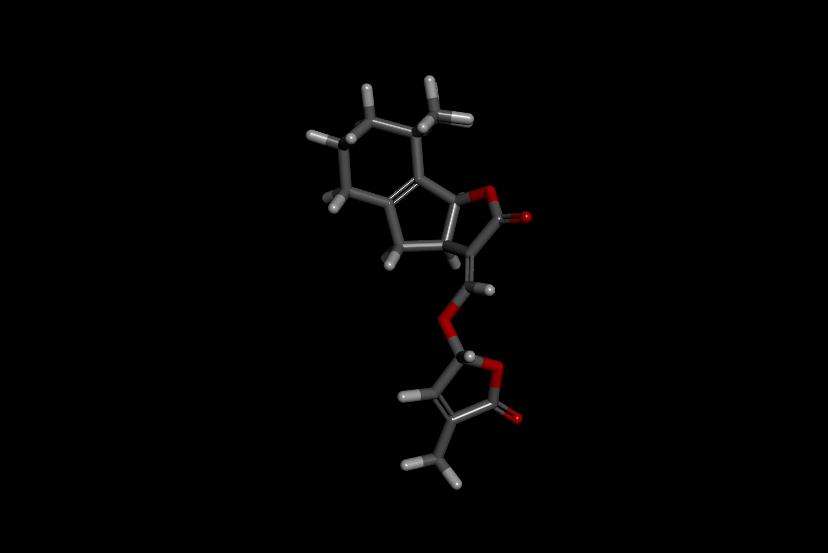


Apo-10'-fucoxanthinal


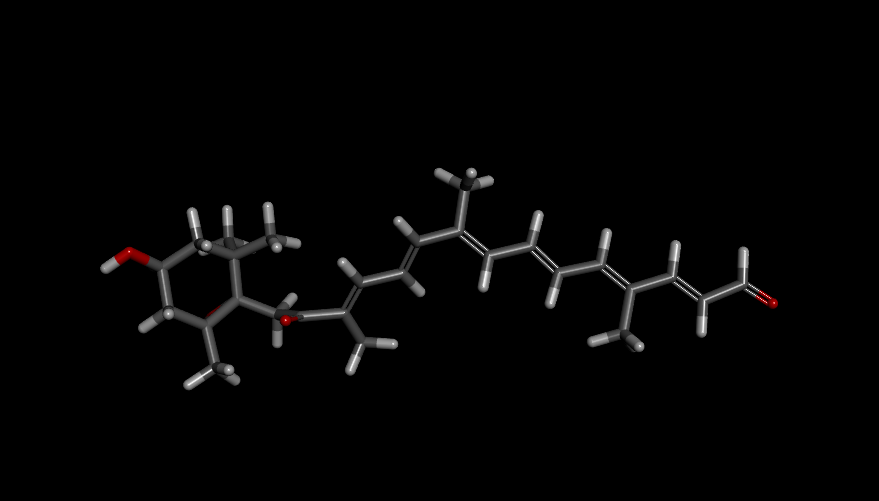


Apo-12'-violaxanthal


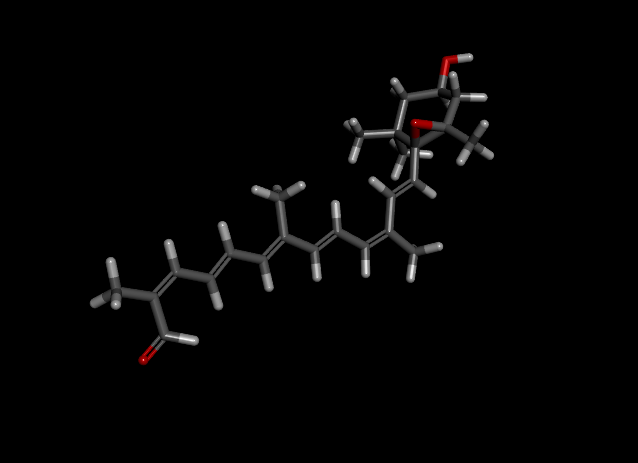


F348


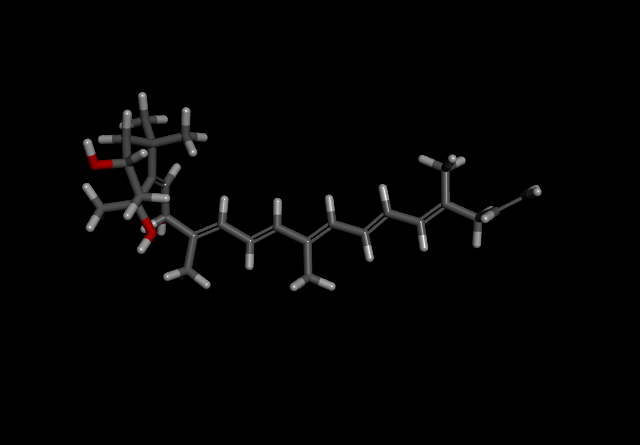


Orobanchol


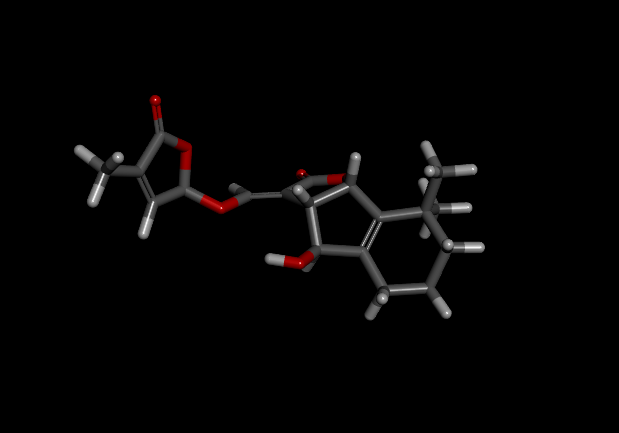


Persicachrome


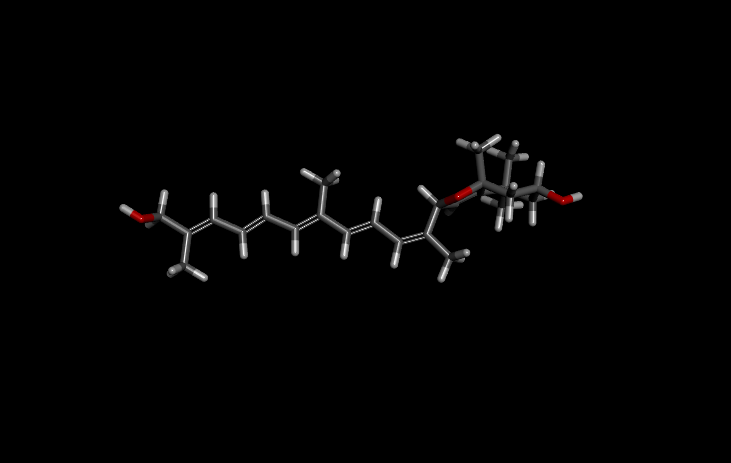


Sinensiaxanthin


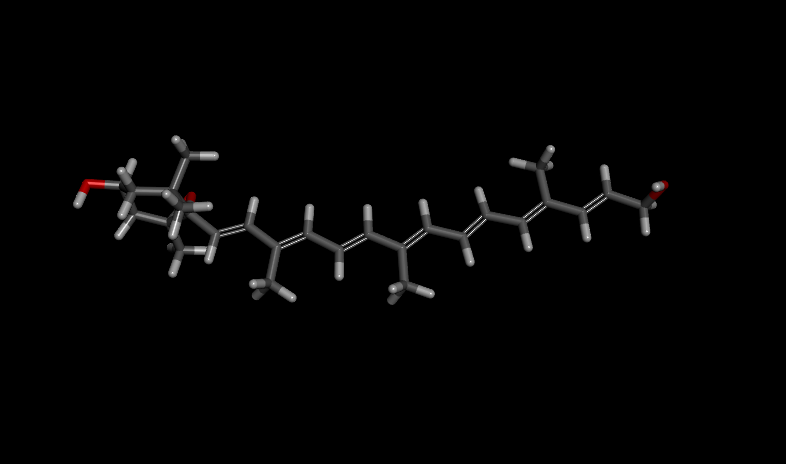


Sorgolactone


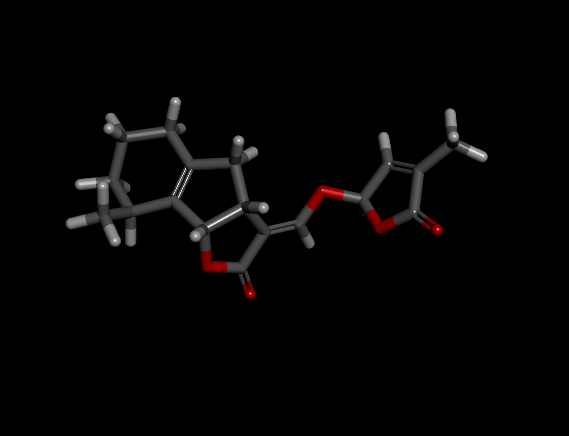


Sorgomol


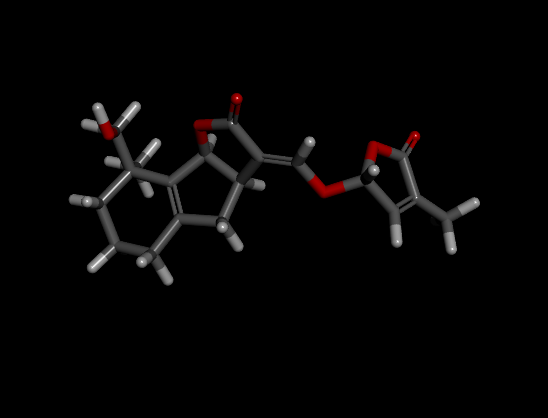


Strigol


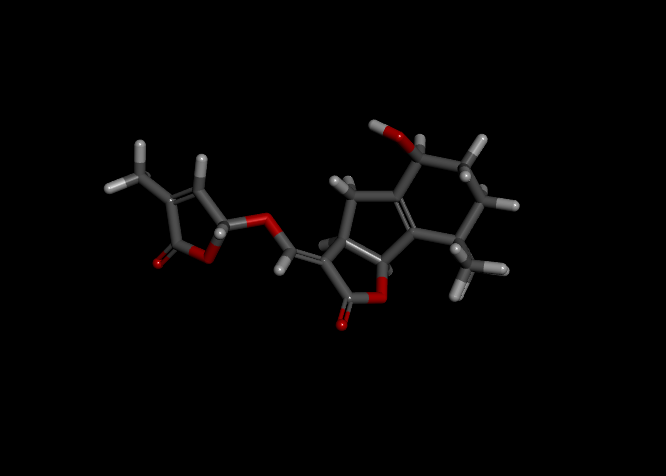


Strigyl acetate


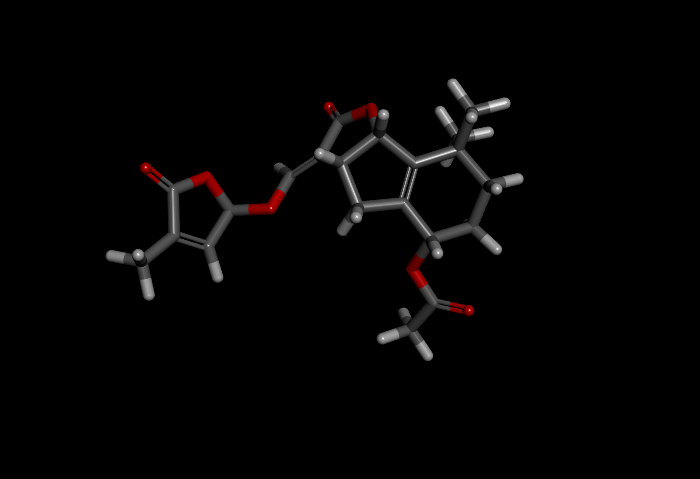


Valenciaxanthin


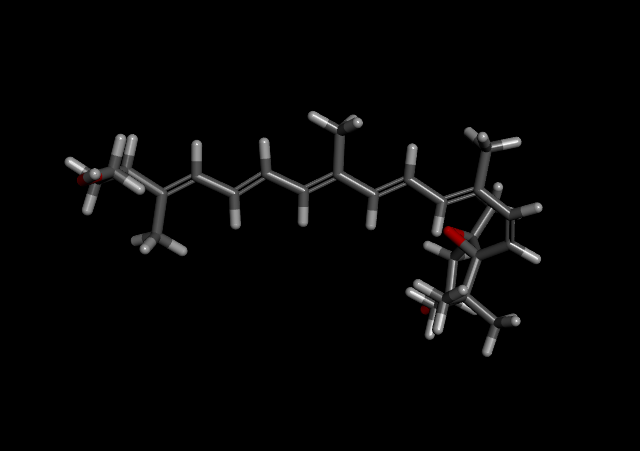

Supplement: S1 File — 13 carotenoids were selected based on docking results and with the most appropriate pharmacokinetics properties. (DOCX) [file pone.0276538.s004.docx]
